# Epidemiology of Intestinal Polyparasitism among Orang Asli School Children in Rural Malaysia

**DOI:** 10.1371/journal.pntd.0003074

**Published:** 2014-08-21

**Authors:** Ahmed K. Al-Delaimy, Hesham M. Al-Mekhlafi, Nabil A. Nasr, Hany Sady, Wahib M. Atroosh, Mohammed Nashiry, Tengku S. Anuar, Norhayati Moktar, Yvonne A. L. Lim, Rohela Mahmud

**Affiliations:** 1 Department of Parasitology, Faculty of Medicine, University of Malaya, Kuala Lumpur, Malaysia; 2 Department of Community Medicine, Faculty of Medicine, University of Al-Anbar, Al-Anbar, Iraq; 3 Department of Parasitology, Faculty of Medicine and Health Sciences, Sana'a University, Sana'a, Yemen; 4 Institute of Medical Molecular Biotechnology, Faculty of Medicine, University Teknologi MARA, Jalan Hospital, Sungai Buloh, Selangor, Malaysia; 5 Department of Medical Laboratory Technology, Faculty of Health Sciences, Universiti Teknologi MARA, Selangor, Malaysia; 6 Department of Parasitology and Medical Entomology, Faculty of Medicine, Universiti Kebangsaan Malaysia, Jalan Raja Muda Abdul Aziz, Kuala Lumpur, Malaysia; Hitit University, Turkey

## Abstract

**Background:**

This cross-sectional study aimed to investigate the current prevalence and risk factors associated with intestinal polyparasitism (the concurrent infection with multiple intestinal parasite species) among Orang Asli school children in the Lipis district of Pahang state, Malaysia.

**Methods/Principal findings:**

Fecal samples were collected from 498 school children (50.6% boys and 49.4% girls), and examined by using direct smear, formalin-ether sedimentation, trichrome stain, modified Ziehl Neelsen stain, Kato-Katz, and Harada Mori techniques. Demographic, socioeconomic, environmental, and personal hygiene information were collected by using a pre-tested questionnaire. Overall, 98.4% of the children were found to be infected by at least one parasite species. Of these, 71.4% had polyparasitism. The overall prevalence of *Trichuris trichiura*, *Ascaris lumbricoides*, hookworm, *Giardia duodenalis*, *Entamoeba* spp., and *Cryptosporidium* spp. infections were 95.6%, 47.8%, 28.3%, 28.3%, 14.1% and 5.2%, respectively. Univariate and multivariate analyses showed that using an unsafe water supply as a source for drinking water, presence of other family members infected with intestinal parasitic infections (IPI), not washing vegetables before consumption, absence of a toilet in the house, not wearing shoes when outside, not cutting nails periodically, and not washing hands before eating were significant risk factors associated with intestinal polyparasitism among these children.

**Conclusions/Significance:**

Intestinal polyparasitism is highly prevalent among children in the peninsular Malaysian Aboriginal communities. Hence, effective and sustainable control measures, including school-based periodic chemotherapy, providing adequate health education focused on good personal hygiene practices and proper sanitation, as well as safe drinking water supply should be implemented to reduce the prevalence and consequences of these infections in this population.

## Author Summary

Intestinal parasitic infections (IPI) are still a major public health problem worldwide, with more than 2 billion people infected with at least one parasite species. Despite efforts to improve the quality of life of the Orang Asli population in rural Malaysia, IPI are still highly prevalent and of serious concern in this population, especially among children. We screened 498 school children in Lipis district, Pahang, Malaysia for the prevalence and risk factors of polyparasitism (concurrent infection with multiple parasite species). Overall, 98.4% of the children were found to be infected by at least one parasite species, with 71.4% of them having polyparasitism. Using an unsafe water supply as a source for drinking water, presence of another family member infected with IPI, not washing vegetables before consumption, absence of a toilet in the house, not wearing shoes when outside, not cutting nails periodically, and not washing hands before eating were significantly associated with intestinal polyparasitism among these children. Our findings revealed an urgent need to implement an effective and integrated control program to reduce the prevalence of IPI as a part of the efforts to improve the quality of life in Orang Asli communities.

## Introduction

Intestinal parasitic infections (IPI) are still public health problems in many communities, particularly among children in rural areas of developing countries. It is estimated that more than 2 billion people worldwide are infected with IPI and more than half of the world's population are at risk of infection [1], [2]. These infections are caused by helminth parasites such as soil-transmitted helminths (*Ascaris lumbricoides*, *Trichuris trichiura*, *Strongyloides stercoralis*, and hookworm), *Taenia* spp. and *Hymenolepis nana* or by protozoa such as *Entamoeba histolytica*, *Giardia duodenalis*, and *Cryptosporidium* spp.

IPI are associated with high morbidity particularly among young children and women of childbearing age, and have been termed as ‘the cancers of developing nations’ by Egger et al. [3]. IPI can occur in silence as chronic infections and infected individuals are either asymptomatic or suffering from mild diseases. However, acute and severe IPI, especially with pathogenic *Entamoeba* and *Giardia*, may cause fatal diarrhea especially among children and both are commonly associated with travellers' diarrhea [4], [5]. Moreover, *Entamoeba* can cause invasive intestinal infection or disseminate to the liver (and rarely to the lung and the brain) causing amebic liver abscess with about 100,000 deaths annually, making amebiasis the second leading cause of death from protozoal diseases, after malaria [6], [7]. On the other hand, opportunistic IPI such as *Cryptosporidium*, *Isospora belli*, Microsporidia, and *Strongyloides* infections are commonly reported among immunocompromised individuals with significant morbidity and mortality [8], [9].

There is a general acceptance that severe IPI are likely to result in failure to thrive and poor growth in children [10]–[12], vitamin A deficiency [13], [14], iron deficiency anemia [15], [16], and poor educational performance [17], [18]. Moreover, recent studies highlighted the impact of polyparasitism on the hosts' immunity and showed that polyparasitism is associated with higher mortality rates and may increase the susceptibility to other infections relative to infection with a single parasite [19]–[21].

In Malaysia, despite sustained socioeconomic and infrastructural development, IPI are still highly prevalent, especially among impoverished rural communities. Several previous studies have investigated the prevalence of IPI and reported the findings either on a specific parasitic infection, monoparasitism [22]–[24], or groups of parasitic infections such as soil-transmitted helminths (STHs) and intestinal protozoa [25]–[29], or on the overall prevalence of IPI [30], [31]. These studies revealed high prevalence rates of IPI with prominent morbidity among Orang Asli and other rural populations in Malaysia. However, there has been limited information on polyparasitism among these populations. The present study was, therefore, carried out to determine the prevalence of polyparasitism and to investigate its associated risk factors among Orang Asli school children in Lipis district, Pahang, Malaysia by comparing children who were infected with multiple parasites with those who had only single parasitic infection or were not infected at all.

## Materials and Methods

### Ethics Statement

The present study was carried out according to the guidelines laid down in the Declaration of Helsinki and all procedures involving human subjects were approved by the Medical Ethics Committee of the University of Malaya Medical Centre, Malaysia (reference number: 932.7). Permission was also obtained from the Department of Orang Asli Development (reference number: JHEOA.PP.30.052 Jld. 6) and the Department of Education, Pahang [reference number: JPNP.SPS.UPP.600-2/6(80)]. Before the commencement of the study, meetings were held with the heads of villages, headmasters, and teachers of both schools to provide information about the objectives and protocol of the study and their consents were obtained. During fieldwork, the purpose and procedures of the study were explained to the children and their parents/guardians. Moreover, they were informed that their participation was voluntary and they could withdraw from the study at any time without citing any reason whatsoever. Written and signed or thumb-printed informed consent was obtained to conduct the study from parents or guardians on behalf of their children before starting the survey, and these procedures were approved by the Medical Ethics Committee of the University of Malaya Medical Centre. All the infected children were treated with a 3-day course of 400 mg albendazole. Each child chewed the tablets with chocolate flavoured biscuits before swallowing them while being observed by a researcher and medical officer (direct observed therapy) [2]. Albendazole is considered as the drug of choice for *Ascaris*, *Trichuris*, and hookworm infections and it is also effective for *Giardia* infection [24]. Moreover, all results were submitted to relevant authorities for further follow-up.

### Study Area

This cross-sectional study was carried out between January and April 2012, in the Lipis district of Pahang state, located at the center of Peninsular Malaysia, about 200 km northeast of Kuala Lumpur. The climate is equatorial with hot-humid conditions and rainfall throughout the year. Most of the houses in the area are made of wood or bamboo with inadequate sanitary facilities. More than half of the houses had no piped water supply. However, the villages are located alongside rivers which are the main source of water for daily activities. The majority of the people in the villages work as labourers in palm oil and rubber plantation or are engaged in the selling of forest products, to earn a living.

**Figure 1:**
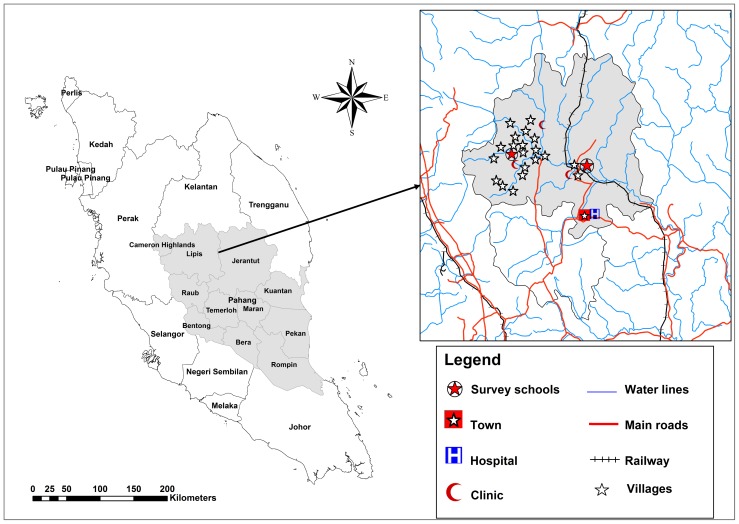
A geographic map showing Pahang state and location of the selected schools and villages in Lipis district.

This study was conducted in two Orang Asli areas namely Kuala Koyan (two villages) and Pos Betau (18 villages) in Lipis district (Figure 1). There are two primary schools at these areas namely, Sekolah Kebangsaan Kuala Koyan (SKKK) and Sekolah Kebangsaan Pos Betau (SKPB). The villages and schools were selected from the available official village list in collaboration with the Department of Orang Asli Development (JAKOA). The selection criteria were as follows: (i) school located in a rural area, (ii) easy access from the main roads, and (iii) school enrolment of more than 100 pupils.

### Study Population

Orang Asli translated as ‘original or first people’ are the Aboriginal minority peoples of Malaysia; representing 0.7% of the country's total population. Although the total enrolment of the schools was 783 pupils (167 SKKK and 616 SKPB), only 650 were present during sampling visits. Of these children who were present, 498 children aged 6–12 years (252 boys and 246 girls) had agreed voluntarily to participate in this study and had met the inclusion criteria (written consent signed by the guardian, completed questionnaire and delivered stool samples for examination).

**Figure 2:**
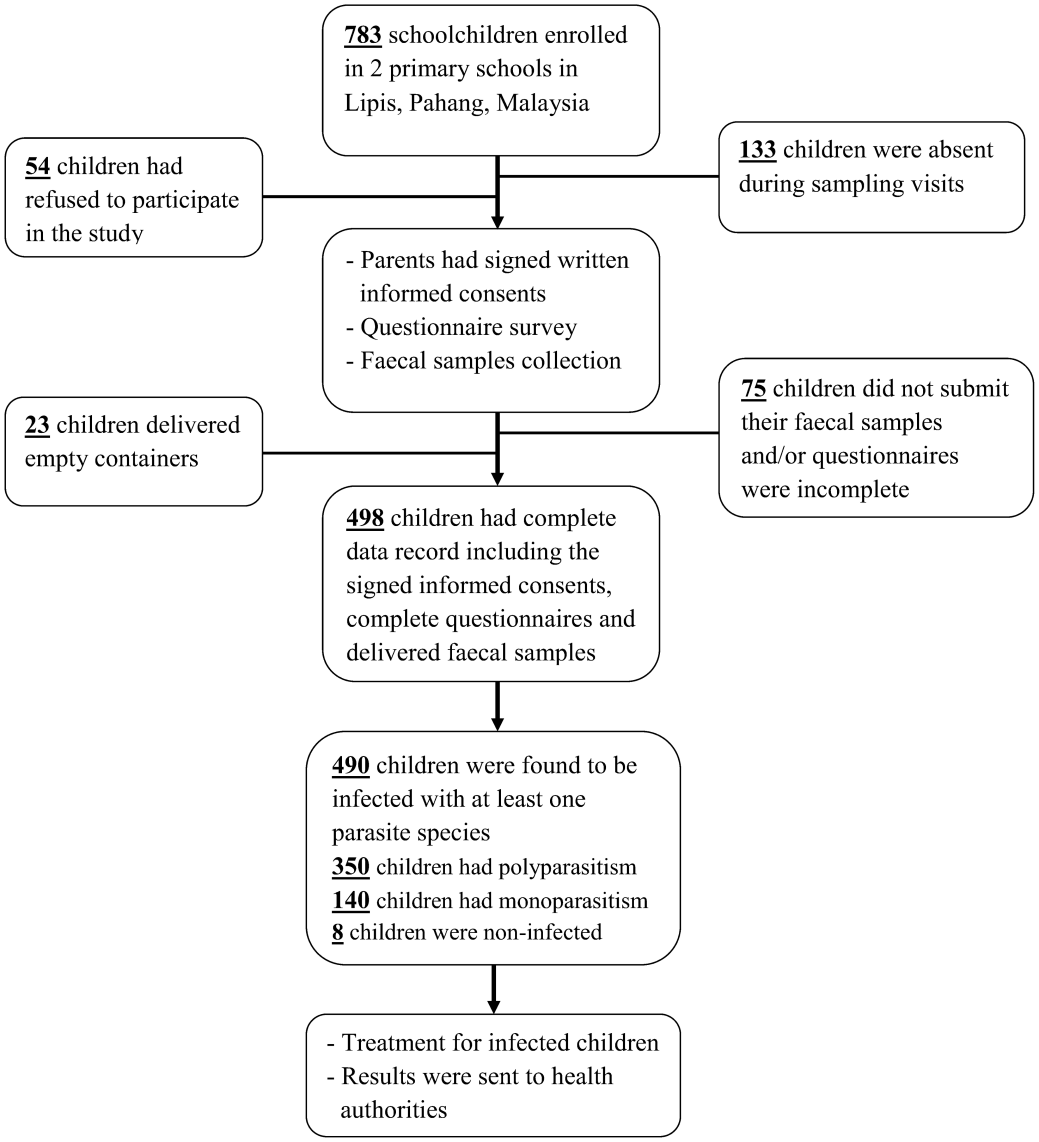
Flow chart of the participation in the present study.

The minimum sample size required for this study was calculated according to the formula provided by Lwanga and Lemeshow [32]. At a 5% level of significance and a 95% confidence level, the minimum number of participants required for the study was estimated at 288, assuming that the prevalence of intestinal parasitic infection among Orang Asli children was about 75% as previously reported [28], [30]. The study flow chart and the participation of children are shown in Figure 2.

### Questionnaire Survey

The demographic, socioeconomic and environmental information, personal hygiene practices, history of receiving anthelmintic treatment and health status of the participants were collected by using a pre-tested questionnaire. The questionnaire was designed in English and then translated into Malay. Two research assistants from the Department of Parasitology, University of Malaya were trained for the purpose of this study on how to administer the questionnaire. The children and their parents were interviewed in their home settings. During the interviews, observations were made on the personal hygiene of the children (e.g., cutting fingernails, wearing shoes when outside the house, and hands and clothes cleanliness), household cleanliness and the availability of functioning toilets and piped water.

### Collection and Examination of Fecal Samples

The children were given a clearly labeled, wide mouth and screw-caps containers and were instructed to bring their early morning stool samples the next day. The collected samples were transported (within 5 hours of collection) in suitable cool boxes at temperature between 4 and 6°C for examination at the stool processing laboratory in the Department of Parasitology, Faculty of Medicine, University of Malaya. The samples were examined by using six different techniques; namely, direct smear, formalin-ether sedimentation, Kato-Katz, Harada Mori, trichrome stain, and modified Ziehl Neelsen stain techniques.

All the samples were screened first by direct smear technique. Then, formalin-ether sedimentation technique was used to increase the detection rates, especially when the samples were negative by direct smear [33]. For the estimation of intensity of STH infections, egg counting was done by using the Kato-Katz technique [2]. Harada Mori culture technique was done to detect hookworm larvae in light infections as described elsewhere [34]. The larvae were collected and examined to distinguish between hookworm and *S. stercoralis* by the characteristic morphology of the larvae (i.e., size of buccal cavity and presence of genital primordium (rhabditiform larvae) or presence of notched tail (filariform larvae). For STH infections, positive samples were recorded according to species and the intensity of infection was recorded as eggs per 1 g of stool (EPG) and was graded as heavy, moderate, or light according to the criteria proposed by the World Health Organization [2].

A suitable amount (approximately 10 g) of faeces was mixed thoroughly and fixed in polyvinyl alcohol (PVA) for the detection of intestinal protozoa (*Giardia* and *Entamoeba*) using trichrome staining technique [35]. Moreover, fecal smears were prepared and stained with modified Ziehl Neelsen stain, according to Henriksen and Pohlenz [36], for the detection of *Cryptosporidium* oocysts. Overall, the samples were considered as positive if the eggs/cysts/trophozoites/oocysts were detected using at least one of these techniques. For quality control, duplicate slides were prepared from 20% of the samples for each diagnostic technique and the slides were read by two different microscopists.

### Data Analysis

Data were reviewed and double-checked before and after data entry by two different researchers. Only those participants who had complete data records, including results of intestinal parasites by the different methods and complete questionnaire, were retained for the final analyses. For descriptive analysis, prevalence of infections and illnesses was expressed in percentage, while mean (standard deviation; SD) or median (interquartile range; IQR) was used to present the quantitative data and results were presented in tables. All quantitative variables were examined for normality by Kolmogorov-Smirnov Z test before analysis. To assess polyparasitism, a variable termed “infection status” was created to denote conditions of non-infected, monoparasitism (infection with a single parasite species), or polyparasitism (co-infections with two or more parasite species).

Pearson's χ^2^ test was used to investigate the association between polyparasitism as the dependent variable and demographic factors (age, gender, and household size), socioeconomic factors (parents' educational and employment status, family monthly income, source of drinking water, presence of toilet in the house, having domestic animals in the households, and presence of infected family member), and personal hygiene practices (washing hands before eating and after defecation, washing fruits and vegetables before consumption, wearing shoes when outside, eating soil (geophagy), boiling drinking water, cutting nails periodically, and indiscriminate defecation) as explanatory variables. All variables in the survey were coded in a binary manner as dummy variables. For example, polyparasitism (positive = 1, negative = 0); gender (boys = 1, girls = 0); presence of toilet in the house (no = 1, yes = 0), and washing vegetables before eating (no = 1, yes = 0). Family size was categorized into two groups (≥7 and <7 members), and age of participants was categorized into two groups that were below 10 years and ≥10 years according to previous studies conducted among Orang Asli [13], [24], [28]. Odd ratios (ORs) and 95% confidence intervals (CIs) were computed for all variables. To adjust for multiple comparisons, we calculated critical significance thresholds for each factors group using the sequential Bonferroni correction [37].

Multivariable logistic regression model was performed to identify risk factors that were significantly associated with intestinal polyparasitism; coded as 1 = polyparasitism, 0 = mono-parasitism and uninfected. In order to retain all possible significant associations, variables that showed an association with *P*≤0.25 were used in the logistic regression model, as suggested by Bendel and Afifi [38]. Moreover, gender variable was also included in the multivariable analysis as it has been considered as an important behavioral modifying factor [39]. Overall, 15 variables met our inclusion criteria to the final model. Many explanatory variables were included separately in this study as there is limited preceding evidence to support inclusion of one factor before or instead of others. Moreover, the different personal hygiene practices are included separately in order to reflect distinct parasite transmission routes. Population attributable risk fraction (PARF) was calculated for significantly associated risk factors [40]. Data analysis was done by using SPSS for WINDOWS (version 13.0; SPSS Inc, Chicago, IL), and significance was set at *P*<0.05.

## Results

### General Characteristics of Participants

The demographic and socioeconomic characteristics of the participants are shown in Table 1. Four hundred and ninety eight school children aged between 6 and 12 years, with a median age of 9 years (IQR 8–11 years) had participated in this study. The children were from 20 villages in Lipis, Pahang. Of these children, 50.6% were boys and 49.4% were girls. In general, poverty prevails in these communities and about two thirds of the families had low monthly income (<RM 500, 156 US$); the poverty income threshold in Malaysia [41]. Moreover, about half and 40.2% of the mothers and fathers, respectively, had no formal education. A majority of the mothers and two thirds of the fathers are not working. Those working were mainly engaged in agriculture (rubber and oil palm plantations), forestry, fishing, and related occupations. Almost half of the houses are without toilets and it was found that Orang Asli people preferred to defecate at the site of the streams. The data from the questionnaire survey indicating none of the children had received anthelmintic treatment in the 6 months prior to the study.

**Table 1:** General characteristics of Orang Asli children who participated in the study (n = 498).

Characteristics	n (%)
**Age groups**	
<10 years	290 (58.2)
≥10 years	208 (41.8)
**Gender**	
Boys	252 (50.6)
Girls	246 (49.4)
**Socioeconomic status**	
Fathers' education level (at least primary)	298 (59.8)
Mothers' education level (at least primary)	249 (50.0)
Low household income (<RM 500)	317 (63.7)
Working fathers	176 (35.3)
Working mothers	66 (13.3)
Large family size (≥7 members)	247 (49.6)
Piped water supply	243 (48.8)
Electricity	364 (73.1)
Presence of toilet in house	231 (46.4)
Presence of domestic animals at household	356 (71.5)

All values are number (%). RM, Malaysian Ringgit; (US$ 1 = RM 3.20).

### Prevalence and Distribution of Intestinal Parasitic Infections

Fecal samples were screened by different techniques for the presence of intestinal parasites and the results are presented in Table 2. Overall, 98.4% (490/498) of the children were found to be infected with at least one intestinal parasite species. The results showed that *T. trichiura* was the predominant species with a prevalence rate of 95.6% (476/498), followed by *A. lumbricoides* (47.8%), *G. duodenalis* (28.3%), and hookworm (27.9%). The results also showed that almost two thirds and 62.0% of the *T. trichiura* and *A. lumbricoides* infections, respectively, were of moderate-to-heavy intensity (mean EPG faeces of ≥5,000 for *A. lumbricoides* and ≥1,000 for *T. trichiura*), while all hookworm infections were of light intensity (≤2,000 EPG). The detection rates of *T. trichiura*, *A. lumbricoides*, hookworm, *G. duodenalis* and *E. histolytica/E. dispar* by formalin-ether sedimentation method were found to be 93.4%, 46.6%, 21.9%, 25.9% and 12.0%, respectively. On the other hand, lower detection rates were noted with Kato-Katz technique; *T. trichiura* (86.7%), *A. lumbricoides* (45%) and hookworm (10.6%). However, the detection rate of hookworm was substantially higher (22.5%) by using Harada Mori technique while almost similar detection rates for *G. duodenalis* (23.5%) and *E. histolytica/E. dispar* (11.4%) were observed by using trichrome staining technique when compared with the results obtained by the formalin-ether sedimentation method.

**Table 2:** Prevalence of intestinal parasitic infections according to parasite species and number of infections.

Infections	No. positive	%
**Parasite species (n = 498)**		
*Trichuris trichiura*	476	95.6
*Ascaris lumbricoides*	238	47.8
Hookworm	139	27.9
*Giardia duodenalis*	141	28.3
*Entamoeba* spp.	70	14.1
*Cryptosporidium* spp.	26	5.2
**Type of infection (n = 490)**		
Monoparasitism	140	28.6
Polyparasitism	350	71.4
**No. of parasite species (n = 350)**		
Two	189	54.0
Three	88	25.1
Four	54	15.4
Five	19	5.4

**Figure 3:**
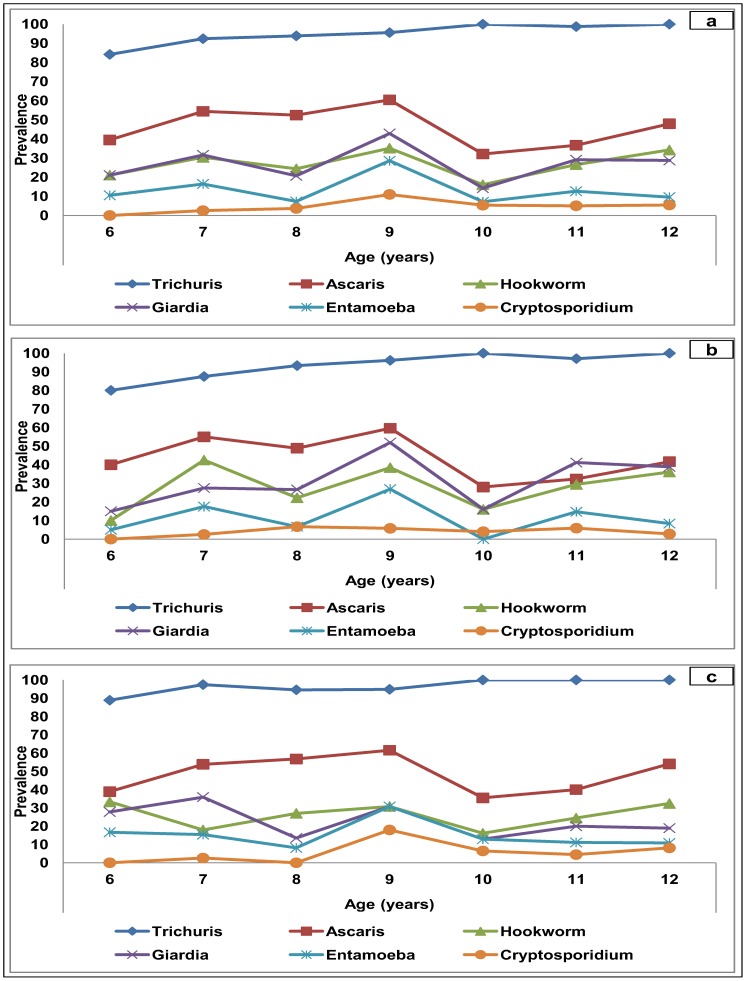
Age-associated prevalence of intestinal parasitic infections among Orang Asli children in Lipis, Pahang (n = 498). a: Overall. b: Boys. c: Girls.

The age-associated prevalence of different reported IPI is illustrated in Figure 3. The age- associated patterns of infection prevalence were generally similar among the different parasite species. *T. trichiura* was the most prevalent infection in all ages followed by *A. lumbricoides* and *G. duodenalis* infection. All IPI, except *Cryptosporidium*, occurred in all ages with the highest prevalence noted among children aged 9 years. When we compared the results according to gender, boys were noted to have higher prevalence of hookworm and *G. duodenalis* while other IPI were higher among the girls. However, the differences were not statistically significant (statistical analysis not shown). On the other hand, *Cryptosporidium* was not reported among children aged 6 years and the highest prevalence was reported among girls aged 9 years (17.9%).

Of the infected children, 71.4% had polyparasitism, while the remaining 28.6% were infected with a single parasite species. Of those who had polyparasitism, 189 (54.0%) and 88 (25.1%) had double and triple infections, respectively. Moreover, 5.4% of them harbored five different parasite species concurrently. *T. trichiura* and *A. lumbricoides* were the most prevalent co-infection representing 54.0% of the polyparasitism prevalence, followed by the combination of *T. trichiura* and *G. duodenalis* (28.4%). Moreover, 18.6% of the infected children had the three STH species (*T. trichiura*, *A. lumbricoides* and hookworm). Fecal samples were also examined for the presence of other intestinal parasites and the children were found to be positive for *Entamoeba coli* (15.5%), *Blastocystis* sp. (15.1%), *Iodamoeba bütschlii* (6.8%), *Chilomastix mesnili* (4.8%), and *Endolimax nana* (2.6%). On the other hand, *S. stercoralis* larvae were not detected among these children.

### Risk Factors of Polyparasitism

Table 3 displays the associations of polyparasitism with demographic, socioeconomic, environmental and personal hygiene factors. The results showed that the prevalence of polyparasitism was significantly higher among children aged <10 years (73.8%) when compared with those aged ≥10 years (65.4%). With regard to socioeconomic factors, children born to non-educated mothers (i.e. <6 years formal education) had significantly higher prevalence of polyparasitism (76.3%) as compared to those of educated mothers (64.3%). It was also found that polyparasitism was higher among children from families with low household monthly income (<RM 500) (74.4%) when compared with children who belong to families with higher household monthly income (63.0%). The results further showed that children who live in houses without toilets (80.1%) and those who use unsafe sources for drinking water (80.2%) had higher prevalence of polyparasitism when compared to those who live in houses with functioning toilets (61.8%) and those who use piped water (60.8%).

**Table 3:** Univariate analysis of factors associated with polyparasitism among Orang Asli children in Lipis, Pahang (n = 498).

Variables	Polyparasitism		OR (95% CI)	*P* value
	No. examined	Infected n (%)		
**DEMOGRAPHIC FACTORS**				
**Age**				
<10 years	290	214 (73.8)	1.52 (1.03, 2.24)	0.043
≥10 years	208	136 (65.4)	1	
**Gender**				
Boys	252	173 (68.7)	0.57 (0.52, 1.33)	0.420
Girls	246	177 (72.0)	1	
**Family size**				
≥7 members (large)	247	170 (68.8)	0.87 (0.61, 1.31)	0.481
<7 members	251	180 (71.7)	1	
**SOCIOECONOMIC FACTORS**				
**Father's educational level**				
Non educated	200	146 (73.0)	1.23 (0.81, 1.87)	0.277
Educated (≥6 years)	298	204 (68.5)	1	
**Mother's educational level**				
Non educated	249	190 (76.3)	1.76 (1.23, 2.58)	0.003*
Educated (≥6 years)	249	160 (64.3)	1	
**Father's employment status**				
Not working	322	223 (69.3)	0.86 (0.59, 1.27)	0.498
Working	176	127 (72.2)	1	
**Mother's employment status**				
Not working	432	306 (70.8)	1.18 (0.67, 2.12)	0.490
Working	66	44 (66.7)	1	
**Household monthly income**				
<RM 500	317	237 (74.4)	1.77 (1.23, 2.66)	0.004*
≥RM 500	181	115 (63.0)	1	
**Presence of toilet in house**				
No	231	185 (80.1)	2.47 (1.67, 3.67)	<0.001*
Yes	267	165 (61.8)	1	
**Presence of domestic animals**				
Yes	356	256 (71.9)	1.27 (0.89, 1.98)	0.208
No	142	94 (66.2)	1	
**Source of drinking water**				
Unsafe source (river, rain)	243	195 (80.2)	2.63 (1.76, 3.88)	<0.001*
Safe source (pipe)	255	155 (60.8)	1	
**Presence of infected family member**				
Yes	207	166 (80.2)	2.42 (1.57, 3.62)	<0.001*
No	291	184 (63.2)	1	
**PERSONAL HYGIENE FACTORS**				
**Washing hands before eating**				
No	297	228 (76.8)	2.13 (1.46, 3.22)	<0.001*
Yes	201	122 (60.7)	1	
**Washing hands after defecation**				
No	296	220 (74.3)	1.64 (1.08, 2.37)	0.017
Yes	202	130 (64.4)	1	
**Indiscriminate defecation**				
Yes	336	240 (71.4)	1.23 (0.78, 1.81)	0.420
No	162	110 (67.9)	1	
**Habit of eating soil (Geophagy)**				
Yes	125	93 (74.4)	1.28 (0.82, 2.09)	0.244
No	373	257 (68.9)	1	
**Cutting nails periodically**				
No	267	207 (77.5)	2.14 (1.38, 3.12)	<0.001*
Yes	231	143 (61.9)	1	
**Wearing shoes when outside**				
No	240	181 (75.4)	1.63 (1.12, 2.44)	0.016
Yes	258	169 (65.5)	1	
**Washing fruits before eating**				
No	262	214 (81.7)	3.28 (2.17, 4.87)	<0.001*
Yes	236	136 (57.6)	1	
**Washing vegetables before eating**				
No	262	211 (80.5)	2.86 (1.86, 4.32)	<0.001*
Yes	236	139 (58.9)	1	
**Boiling water before drinking**				
No	363	252 (69.4)	0.87 (0.56, 1.34)	0.491
Yes	135	98 (72.6)	1	

RM, Malaysian Ringgit; (US$ 1 = RM 3.2). OR, Odds ratio. CI, Confidence interval.

Significant association (unadjusted *P*<0.05).

* Significant association (using the Bonferroni correction for multiple comparisons).

Examining the association of polyparasitism with personal hygiene practices among these children showed that the prevalence of infection was significantly higher among children who do not wash their hands before eating (76.7%) and also after defecation (74.3%), those who walk barefooted (75.4%) and those who do not cut their fingernails periodically (77.5%) when compared to their counterparts. Moreover, children who do not wash fruits and vegetables before consuming had significantly higher prevalence of polyparasitism compared to those who wash the fruits and vegetables before consuming (*P*<0.001). Besides these variables, the presence of other family members infected with IPI was significantly associated with higher rates of polyparasitism among these children (*P*<0.001). Using the Bonferroni adjustment, a series of χ^2^ analyses revealed no association between polyparasitism and age groups, not washing their hands after defecation, and not wearing shoes when outside the house.

Table 4 shows that the multiple logistic regression model identified seven variables as significant risk factors of polyparasitism. Hosmer–Lemeshow test, used for the inferential goodness-of-fit test, showed that the model was fit to the data well (χ^2^ = 9.2; *P* = 0.320). The results of the logistic regression model including all 15 factors confirmed that children who use unsafe sources for drinking water and/or live in houses without proper toilets were at a two times higher odds of having polyparasitism when compared with their counterparts. Likewise, the presence of other family members infected with multiple parasitic infections increased the children's odds for the polyparasitism by 1.7 times.

**Table 4:** Multivariate analysis of factors associated with polyparasitism among Orang Asli children in Lipis, Pahang (n = 498).

Variables	Polyparasitism			
	Adjusted OR	95% CI	Wald	Wald-test P-value
**DEMOGRAPHIC FACTORS**				
Age (<10 years)	1.32	0.75, 2.28	1.463	0.207
Gender (boys)	0.76	0.51, 1.32	0.678	0.410
**SOCIOECONOMIC FACTORS**				
Low mother's educational level (<6 years)	1.23	0.77, 1.87	0.546	0.460
Low household income (<RM 500)	1.44	0.87, 2.28	2.128	0.145
Absence of toilet in house	1.86	1.08, 3.13	5.909	0.014
Presence of domestic animals	1.32	0.67, 2.45	1.451	0.228
Source of drinking water (unsafe water)	2.02	1.31, 3.24	8.871	0.003
Presence of infected family member	1.69	1.07, 2.84	4.937	0.026
**PERSONAL HYGIENE FACTORS**				
Not washing hands before eating	1.63	1.03, 2.62	3.977	0.047
Not washing hands after defecation	1.08	0.67, 1.78	0.135	0.714
Habit of eating soil (Geophagy)	1.42	0.89, 2.48	2.668	0.106
Not cutting nails periodically	1.89	1.18, 2.86	6.656	0.010
Not wearing shoes when outside	1.58	1.02, 2.34	3.853	0.049
Not washing fruits before eating	1.23	0.68, 1.98	0.578	0.447
Not washing vegetables before eating	2.54	1.36, 4.23	11.020	0.001

OR, Odds ratio. CI, Confidence interval.

Significant association (*P*<0.05).

It was found that not washing vegetables before consumption increased children's odds for polyparasitism when compared with always washing vegetables by 2.5 times. Furthermore, children who do not wash their hands before eating had 1.6 times odds while those who do not cut their nails periodically and/or walk barefooted were at twice the odds of polyparasitism when compared with their counterparts. When stratified by age group (<10 and ≥10 years), similar risk factors were identified among both groups.

PARF analysis showed that the number of polyparasitism cases would be reduced by 18.0%, 13.6%, 11.9%, and 6.8% if all children had good standards of personal hygiene; namely, washing vegetables before consumption, washing hands before eating, cutting nails periodically, and wearing shoes when outside the house, respectively. Moreover, 13.5%, 12.1%, and 10.3% of the polyparasitism cases could be reduced when the children in this population had a provision of clean and safe drinking water, had toilet facilities at home, and had no other family members infected with polyparasitism, respectively.

## Discussion

IPI remain major public health problems worldwide particularly among rural children in developing countries. In Malaysia, several previous studies were carried out among rural communities and showed that *T. trichiura*, *A. lumbricoides*, hookworm and *G. duodenalis* infections are highly prevalent [22]–[25], [28]–[30]. These infections have been classified among the most prevalent neglected tropical diseases (NTDs) as they primarily persist in underprivileged communities in remote, rural areas, urban slums or in conflict zones and refugees, and have been largely eliminated elsewhere and thus are often forgotten [1].

The findings of the present study showed that almost all (490/498) of the participating children were positive for at least one parasite species with *T. trichiura* infection being the most common IPI (95.6%) in these children, followed by *A. lumbricoides* and *G. duodenalis* infections. These findings are in agreement with many previous studies conducted among Orang Asli children [25], [27]–[29]. Our findings also showed that 14.1% and 5.2% of the children were infected with *E. histolytica/dispar/moshkovskii* and *Cryptosporidium* spp., respectively, which are consistent with the findings in previous reports [23], [42]. We also found that almost two-thirds and half of the *T. trichiura*, and *A. lumbricoides*, respectively, were of moderate-to-heavy intensities and this level of worm burden is associated with the negative consequences [43]. Since the 1930s, this high prevalence of IPI remained largely unchanged among Aboriginal and rural populations in Malaysia [44]. However, a great reduction of these infections was reported in urban areas [45]. This unchanged trend was found to be closely associated with contaminated environment and poor personal hygiene practices. Moreover, the re-infection rate of IPI after effective treatment was found to be very high and this reveals continuous exposure to the sources of infections in these communities [46], [47].

The findings of the present study showed that the majority of the infected children had polyparasitism (71.4%) while only 28.6% had monoparasitism. The findings further revealed that almost half and a quarter of the polyparasitism were concurrent infections with two and three parasite species, respectively. These findings indicate that the Orang Asli environment is heavily contaminated with the parasites. Moreover, poor knowledge, attitude and practices towards intestinal helminth infections have been reported among this population [48]. Interestingly, 5.4% of the polyparasitized children were infected by five different parasite species (mainly the three STH species, *G. duodenalis* and *E. histolytica/E. dispar*). Similar findings were reported among Kenyan school children with the inclusion of *Schistosoma mansoni* infections [49]. A much higher prevalence of polyparasitism was reported among 500 participants in western Côte d'Ivoire [50]. The study revealed that three quarters of the studied population harbored at least three parasites concurrently, including high prevalence rate of many intestinal commensals such as *Entamoeba coli*, *Blastocystis hominis*, *Entamoeba hartmanni*, *Iodamoeba butschlii*, *Chilomastix mesnili* and *Endolimax nana* and this could explain the very high polyparasitism rate reported among this population. In the present study, high prevalence of *Blastocystis* sp. and other intestinal commensals were also reported. However, the pathogenicity of *Blastocystis* is still controversial and numerous clinical and epidemiological studies concluded that *Blastocystis* is a commensal organism and probably is not responsible for clinical symptoms [51], [52]. Hence, only pathogenic parasites of public health significance were considered in the statistical analysis in order to conclude useful findings and important implications about polyparasitism.

Our findings showed that *T. trichiura*, and *A. lumbricoides* co-infection was the highest (54.0%) and this could be attributed to their common transmission pattern (ingestion of infective eggs), and could be favored by behavioral factors. A previous study suggested a clustering of *A. lumbricoides* and *T. trichiura* infections within households in endemic areas [53]. Similarly, co-infection with *T. trichiura* and *G. duodenalis* was reported among more than one quarter of the polyparasitized children. *T. trichiura*, *A. lumbricoides* and hookworm infections were the most common triple infections (18.6%), followed by *T. trichiura*, *A. lumbricoides* and *G. duodenalis* infections (13.9%). These are important findings since it implies that individuals with polyparasitism may also suffer multiple morbidity due to each parasite species infection. It is also suggested that individuals with multiple species infections are likely to be at highest risk of significant morbidity due to the number of parasite species they harbor and intensity of each infection [54]. Although, *S. stercoralis* was not reported by the present study, a previous study has detected *S. stercoralis* larvae in 7.1% of soil samples collected from the study area (Lipis district) [55]. Out of 54 Orang Asli individuals, 17 were tested seropositive for *Strongyloides*, and then only three of them were confirmed by polymerase chain reaction (PCR) as positives for *S. stercoralis* DNA amplification using fecal samples [56].

It is well documented that STH and *G. duodenalis* infections are significant predictors of malnutrition, iron deficiency anemia, vitamin A deficiency, poor cognitive functions, high rate of school absenteeism, and poor school performance among Orang Asli children [12], [13], [16], [18], [24]. Thereby, this high rate of intestinal polyparasitism will increase the burden of these negative consequences contributing to the overall backwardness, poverty, and low productivity of Orang Asli population. Polyparasitism was found to be significantly associated with being underweight and stunting among school-age children from rural communities in Honduras [57].

Our results also showed that polyparasitism was significantly higher among younger children compared to those aged ≥10 years and this could be attributed to the higher susceptibility of young children and the relatively better personal hygiene among older children. Similar findings were reported among rural populations in Malaysia and elsewhere [30], [50]. Young children are always reported to have poor personal hygiene including geophagia/pica (habit of eating soil/dirt) and they are at a higher exposure to the source of infections than older children [58], [59]. Moreover, our results showed that the age-associated pattern of all IPI prevalence was almost similar among boys and girls. While aggregation of helminth infections was observed among these children, similar pattern was also noted with intestinal protozoa infections. For all six infections reported by the present study, parasite aggregation (number of parasite species present in each individual) was higher among the 9-year-old children than among their older counterparts. Similar findings were reported elsewhere [60], [61].

This is the first study to provide data on the risk factors of intestinal polyparasitism among Orang Asli in rural Malaysia. Our findings showed that polyparasitism was more likely to occur in children living in houses without safe piped water supply and/or functioning toilets. This is in agreement with the findings in previous reports [62]–[64]. Recent studies among Orang Asli communities reported significantly higher prevalence of STH and *G. duodenalis* infections among those who use untreated sources for drinking water [29], [65]. Orang Asli prefer to live close to rivers which are considered essential for their life. They collect water from the rivers for different purposes including drinking and cooking. Moreover, rivers are also their preferred sites for defecation especially children [28]. Thus, water collected from the rivers is always likely to be contaminated with different parasite species and is considered a source of infection. Our study also identified the presence of another family member infected with IPI as a significant risk factor of polyparasitism. Previous studies showed similar associations with *G. duodenalis* and *E. histolytica/E. dispar* infections among Orang Asli [23], [65]. This may indicate the high transmission occurring within the family as any family member may contract the infection and then serve as a source of infection.

With regard to personal hygiene practices, we found that not washing hands before eating, not cutting nails periodically, walking barefooted, and not washing vegetables before consumption were significant risk factors of polyparasitism among these children. Previous studies among Orang Asli population reported these poor personal hygiene variables as risk factors for STH and giardiasis [27]–[30], [65]. These findings are also consistent with previous reports among children from different countries [29], [66]–[69]. The infective stages could be transmitted to humans by ingestion of the eggs/cyst/oocyst from contaminated food, hands or nails, or skin penetration by the larvae. In the same vein, indiscriminate or open defecation is a common practice in these communities and defecation around the house and play grounds was also observed among young children. In addition, human/animal fecal materials are used as fertilizers in these communities. These practices also enhance the contamination of the environment and are spreading the source of infection, thereby increase the chances of infection and re-infection with IPI. The eggs, larvae, cysts, and oocysts of intestinal parasites can remain viable and infective in the environment (soil/water) for a long period of time.

We also found a significant association between polyparasitism and not washing fruits before eating. However, this association was not retained in the multivariate analysis. Most of the fruits in these communities are tropical peeled fruits like rambutan (*Nephelium lappaceum*), langsat (*Lansium domesticum*), longan (*Dimocarpus longan*), mangosteen (*Garcinia mangostana*), and durian (*Bombaceae durio zibethinus*). However, it was noted that children collected the dropped fruits (rambutan, langsat, longan) from the ground and opened the soft shell by mouth directly without washing or may eat the fruits (durian) using their dirty hands. Thus, fruits could be contaminated with the parasites either from the contaminated ground or from the contaminated hands. Eating fresh fruits were found to be associated with higher risk of giardiasis among Orang Asli children [70]. Based on the PARF results, our findings showed that about half of the polyparasitism cases could be reduced if these children had good standards of personal hygiene while providing toilet facilities and provision of safe drinking water will help in reducing 13.5% and 12.1% of the cases, respectively.

We acknowledge several limitations of our methodology. This study had to rely on a single fecal sample instead of the ideal three consecutive samples because of limitation of resources and the cultural belief of the Orang Asli against giving their fecal samples. Thus, the prevalence rate of parasitic infections is likely to be underestimated due to the temporal variation in egg excretion over hours and days. It is shown in community-based studies that stool examination generally underestimates the prevalence of *S. stercoralis* infection [71]. Moreover, the storage of fecal samples in cold temperature (4–6°C) interfered with the parasitological diagnosis of strongyloidiasis [72]. This may also explain the absence of *Strongyloides* in our study. Although microscopic detection of intestinal protozoa (cysts/oocyst and trophozoites) and helminth (eggs) has been the most widely used diagnostic approach, microscopy is not very sensitive and especially infections of light intensity can be missed when only a single stool sample is analysed [73]. However, examining fecal samples by six different methods could help to overcome this limitation. Many Orang Asli villages are located deep in the jungle with no road access and therefore were not covered by the current study. Higher prevalence rates of intestinal parasitic infections were reported in these areas compared to the villages involved in our study [26]. Orang Asli communities in rural Peninsular Malaysia have almost similar socioeconomic, environmental, and health profiles. Thus, we may speculate that the findings can be generalized to other rural Orang Asli children in other states. However, further investigations are required to confirm these conjectures.

### Conclusions

The findings of the present study reveal that polyparasitism is very common among Orang Asli school children in rural Malaysia. Hence, there is an urgent need to implement an innovative and integrated control program to reduce the prevalence and intensity of these infections significantly and to save these children from the negative impact of IPI as a part of the efforts to improve the quality of life of Orang Asli population. Based on the findings, there is a great need for a proper health education regarding good personal hygiene practices and community mobilization to enhance prevention and instil better knowledge on IPI transmission and prevention in these communities. Moreover, sustainable school-based deworming, providing proper and adequate sanitation and safe water supply should be considered in the control program targeting this population.

## Supporting Information

Checklist S1 STROBE checklist.
(DOC)
